# No carbon limitation after lower crown loss in *Pinus radiata*

**DOI:** 10.1093/aob/mcaa013

**Published:** 2020-01-28

**Authors:** Mireia Gomez-Gallego, Nari Williams, Sebastian Leuzinger, Peter Matthew Scott, Martin Karl-Friedrich Bader

**Affiliations:** 1 New Zealand Forest Research Institute (Scion), 49 Sala Street, Te Papa Tipu Innovation Park, Private Bag 3020, Rotorua, New Zealand; 2 Institute for Applied Ecology New Zealand, School of Sciences, Auckland University of Technology, 31–33 Symonds Street, Auckland, New Zealand; 3 Department of Forest Mycology and Plant Pathology, Swedish University of Agricultural Sciences, 750 07 Uppsala, Sweden; 4 The New Zealand Institute for Plant & Food Research Limited, Private Bag 1401, Havelock North, New Zealand

**Keywords:** *A/C*_i_ curves, defoliation, biomass, growth, leaf area, non-structural carbohydrates, photosynthesis, *Pinus radiata*, root:shoot, foliar pathogens

## Abstract

**Background and Aims:**

Biotic and abiotic stressors can cause different defoliation patterns within trees. Foliar pathogens of conifers commonly prefer older needles and infection with defoliation that progresses from the bottom crown to the top. The functional role of the lower crown of trees is a key question to address the impact of defoliation caused by foliar pathogens.

**Methods:**

A 2 year artificial defoliation experiment was performed using two genotypes of grafted *Pinus radiata* to investigate the effects of lower-crown defoliation on carbon (C) assimilation and allocation. Grafts received one of the following treatments in consecutive years: control–control, control–defoliated, defoliated–control and defoliated–defoliated.

**Results:**

No upregulation of photosynthesis either biochemically or through stomatal control was observed in response to defoliation. The root:shoot ratio and leaf mass were not affected by any treatment, suggesting prioritization of crown regrowth following defoliation. In genotype B, defoliation appeared to impose C shortage and caused reduced above-ground growth and sugar storage in roots, while in genotype A, neither growth nor storage was altered. Root C storage in genotype B decreased only transiently and recovered over the second growing season.

**Conclusions:**

In genotype A, the contribution of the lower crown to the whole-tree C uptake appears to be negligible, presumably conferring resilience to foliar pathogens affecting the lower crown. Our results suggest that there is no C limitation after lower-crown defoliation in *P. radiata* grafts. Further, our findings imply genotype-specific defoliation tolerance in *P. radiata*.

## INTRODUCTION

Forest productivity and resilience are increasingly influenced by global change-driven biotic and abiotic factors. Pathogens, insects, drought and any combination thereof have the potential to cause forest decline through different pathways (McDowell *et al.*, 2011; Anderegg *et al.*, 2015). The main consequences of those stressors are progressive growth reduction and defoliation, which have been linked to forest decline and tree mortality and used as predictors for tree death (Dobbertin and Brang, 2001; Bréda *et al.*, 2006; Carnicer *et al.*, 2011; Cailleret *et al.*, 2017). Due to its dramatic consequences for forest functioning and stability, drought-induced defoliation and tree mortality have been well studied over the last decade (Galiano *et al.*, 2011; Allen *et al.*, 2015; Adams *et al.*, 2017). Defoliation events caused by insects have also been given considerable attention, e.g. bark beetle outbreaks, alone or in interaction with drought (Gaylord *et al.*, 2013; Anderegg *et al.*, 2015; Arango-Velez *et al.*, 2016), and leaf-feeding insects (Krause and Raffa, 1992; Quentin *et al.*, 2010; Eyles *et al.*, 2011*a*; Stone *et al.*, 2013; Chen *et al.*, 2017). However, there is a remarkable lack of studies on the impact of pathogen-induced defoliation on tree productivity and resilience through mechanistic approaches despite persistent increases in invasive pathogen alerts and predictions of future increases (Anderson *et al.*, 2004; Fisher *et al.*, 2012; Scott *et al.*, 2019). This knowledge gap challenges the establishment of process-based growth loss models and their inclusion in a wider framework of tree death, accounting for the interaction with other stressors (McDowell *et al.*, 2011; Dietze and Matthes, 2014).

Fungal and oomycete pathogens affect different plant tissues with widely varying impacts. Root rot and stem cankers impair the vascular system and can be the primary cause of tree death. This is the case with the root pathogen *Phytophthora cinammomi* (Shearer *et al.*, 2004), and *Cryphonectria parasitica* causing the chestnut blight (Anagnostakis, 1987). The contribution of defoliation caused by root rot, canker and shoot dieback pathogens, as a predisposing factor to forest decline, has previously been analysed (Aguade *et al.*, 2015; Oliva *et al.*, 2016), but the physiological impact of those pathogens on the tree differs from that of foliar pathogenic infection. Foliar pathogens rarely cause tree death, but result in defoliation and reduced growth rates. Several characteristics of the foliar pathogenic infection make it difficult to extrapolate the results of insect- and drought-induced defoliation studies to the impacts of defoliation by foliar pathogens. In evergreen hosts, foliar pathogen infection and defoliation most frequently affect old leaves in the lower crown, although infection can progress up to the whole crown (Scharpf, 1993; Goheen and Willhite, 2006; Tattar, 2012). Drought-induced defoliation typically starts in the upper crown, where leaves experience more negative water potentials (Scharpf, 1993; Tattar, 2012). Defoliation by leaf-feeding insects may affect the entire crown or be restricted to the upper part, as well as differing in leaf age preference (Krause and Raffa, 1992; Pinkard *et al.*, 2011; Barry and Pinkard, 2013; Jetton and Robison, 2014). Other differences include defence mechanisms and duration of the defoliation, with several weeks of foliar pathogenic infection preceding defoliation, compared with the few minutes that leaf-feeding insects need to eat leaves (Krause and Raffa, 1992).

Few studies have analysed the impact of pathogen-induced defoliation on carbon (C) assimilation and allocation. The impacts of the defoliation caused by Swiss needle cast disease on Douglas fir productivity and resilience have been examined in detail (Manter *et al.*, 2003; Lee *et al.*, 2013; Saffell *et al*., 2013, 2014). This disease has an unusual defoliating pattern, as Swiss needle cast infection affects the top of the crown rather than the bottom (Shaw *et al.*, 2014). Surprisingly, lower-crown defoliation, affecting other than current-year leaves, has not been investigated in previous studies.

Leaves located in upper and outer parts of a tree crown have larger photosynthetic capacity and higher amounts of nitrogen (N) stored in photosynthetic proteins, such as Rubisco and chlorophyll-binding polypeptides, compared with leaves in the lower part or interior of the crown (Leuning *et al.*, 1991; Camm, 1993; Ackerly, 1999; Millard and Grelet, 2010; Bresinsky *et al.*, 2013). The foliage density required to maximize C uptake is commonly far exceeded in tree crowns, suggesting different roles for lower-crown foliage other than contributing to the net gain in C assimilation (Thomas and Sadras, 2001; Hirose, 2005; Körner, 2018). Shaded leaves have been recognized to serve as nutrient and C stores for later resorption by younger leaves, and as a buffer in the case of foliage loss, especially in evergreen conifers (Nambiar and Fife, 1987; Thomas and Sadras, 2001; Millard and Grelet, 2010; Hirose, 2012). The loss of lower-crown foliage is likely to have more impacts through nutrient shortage than through reduced C assimilation.

Typically, plant responses to defoliation seek to compensate for the reduction in leaf area, for example through upregulation of photosynthetic C assimilation. Two mechanisms can take place after defoliation, which determine a possible upregulation of photosynthesis. On the one hand, the light regime of the remaining leaves can change. Reduced light levels, as a function of the leaf’s position within the crown, lead to reduced photosynthetic rates (Ackerly, 1999). On the other hand, defoliation can alter the source:sink relations in a tree. Most frequently, the source:sink ratio decreases following defoliation, leading to compensatory photosynthesis in the remaining leaves (Wareing and Patrick, 1975; Lavigne *et al.*, 2001; Battaglia *et al.*, 2011), although increased C uptake in the remaining leaves does not always take place (Cerasoli *et al.*, 2004; Wiley *et al.*, 2013). This photosynthetic upregulation may be only short term (Eyles *et al.*, 2009, 2011a). In addition, it does not appear to occur when defoliation does not impose C limitations on growth, for example when drought stress is limiting tree growth instead (Pinkard *et al.*, 2011; Quentin *et al.*, 2012). Beyond photosynthetic upregulation, crown formation in the following season can completely compensate for the foliage loss if defoliation is not severe. However, that does not come without a cost. Post-defoliation crown regrowth can represent a sizeable sink competing with C storage and stem growth, hence defoliation may result in transient changes in C allocation (Barnes and Edwards, 1980; Chen *et al.*, 2017). The imposition of C (source) limitation by defoliation is controversial and has not been proven *per se*. Most canopies are saturated in terms of light and photosynthesis (Körner 2018). However, it has been suggested that under severe defoliation, C supply indeed becomes limiting (Wiley and Helliker, 2012; Wiley *et al.*, 2013; Palacio *et al.*, 2014). Further, fast recovery of non-structural carbohydrate (NSC) concentrations in defoliated trees to the levels of non-defoliated trees has been reported (Barnes and Edwards, 1980; Palacio *et al.*, 2012; Saffell *et al.*, 2013; Wiley *et al.*, 2013, 2017*a*; Piper *et al.*, 2015; Puri *et al.*, 2015; Chen *et al.*, 2017). This suggests either the prioritization of storage over growth, or that C becomes non-limiting again, after rapid foliage recovery, and subsequently NSC passively increases. The dynamics of C allocated to growth and storage have direct implications for tree productivity and resilience to future stress episodes. It is crucial to understand the changes in C dynamics and under which defoliation intensities and patterns they take place.

The aim of this study was to examine the impact of lower-crown defoliation on C assimilation and allocation in order to better understand pathogenic impacts on tree physiology. We used repeated artificial defoliation in *Pinus radiata* D. Don grafts to simulate the defoliation pattern associated with the Red needle cast (RNC) disease, a new foliar disease caused by *Phytophthora pluvialis*, whose infection peaks in winter (Dick *et al.*, 2014). The RNC disease typically affects lower-crown foliage other than current-year needles (Dick *et al.*, 2014). We used grafts of two RNC-susceptible genotypes growing in the radiata pine plantation forests in New Zealand. In line with the timing of the RNC disease, we removed 1-year-old and older needles from the bottom half of the crown, during two consecutive winters, to check the effect of both a single and two defoliation events. We hypothesized that (1) the removal of old foliage from the lower crown will not cause pronounced upregulation of photosynthesis due to the lack of change in light conditions for the remaining leaves in the upper crown, and the lack of a strong C sink for growth in winter. Further, we anticipated that (2) tree radial and height growth will be reduced, and the reductions will be larger in grafts that undergo two consecutive defoliation treatments than those experiencing a single defoliation event. We also expected that (3) NSC (soluble sugars and starch) pools will show small and temporary reductions (coinciding with crown development) and will recover after a growing season following defoliation.

## MATERIALS AND METHODS

### Plant material and defoliation treatments

**Fig. 1.**
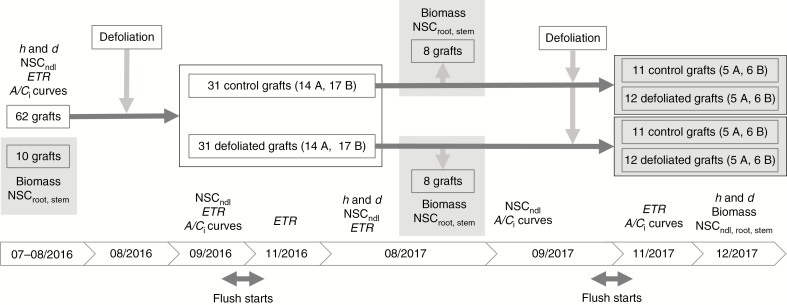
Flowchart illustrating the experimental design, measurements and dates. Shaded areas indicate destructive measurements. NSC_ndl, root, stem_, non-structural carbohydrates sampled from needles, roots and stems, respectively; *ETR*, apparent electron transport rate measured by means of chlorophyll fluorescence; *h*, height; *d*, diameter; A, genotype A; B, genotype B; dates are expressed in mm/yyyy. Unless otherwise specified, the sample size is the same in both genotypes.

A total of 72 *Pinus radiata* D. Don grafts were grown in 45 litre bags under sheltered conditions in the Scion nursery (Rotorua, New Zealand), where they were drip irrigated throughout the experiment. Scions were taken from mature trees and grafted on rootstock from 1-year-old seedlings 2 years before the experiment started. The grafts were ramets of two RNC-susceptible genotypes (genotype A, 33 grafts; genotype B, 39 grafts). The grafts were randomly distributed in three rows (two rows of 2 × 12–13 plants and one more row of 1 × 12 plants), allowing enough space to grow over 2 years without shading effects between plants, i.e. approx. 20 cm between bags within the row and 70 cm between rows. At the beginning of the experiment, the grafts had three whorls, holding 3–4 branches each, and their dimensions were not significantly different between genotypes (averages of 17 mm in diameter, 0.9 m in height and 42 cm in average crown diameter). Ten grafts were harvested for biomass at the beginning of the experiment (Fig. 1). Half of the remaining 62 grafts were artificially defoliated in winter 2016 (August 10–11), mimicking the impact of the RNC disease, while the other half served as the control. In early spring 2017 (September 18–19), 12 of the defoliated grafts underwent a second defoliation. At the same time, 12 of the original controls were defoliated to allow comparison between a single and two consecutive defoliation events. The first-year defoliation consisted of the removal of 75 % of 1-year-old and older needles in the lower half of the crown (repeatedly removing three needles out of four), while in second-year defoliation 100 % of 1-year-old and older needles in the lower-half crown were removed.

### Biomass sampling and growth measurements

Height (*h*, m) and diameter (*d*, mm) at 3 cm up from the graft union were measured on each graft on three occasions: 1 July 2016 (pre-treatment assessment); 1 August 2017 (1 year after the first defoliation); and 15 December 2017 (3 months after second-year defoliation). Pre-treatment data were used to stratify treatments within each of the four height value quartiles, to minimize the effect of initial graft size.

Following growth measurements, sub-samples of ten and 16 grafts were harvested for biomass data on the first and second measurement dates, respectively (Fig. 1). At the end of the experiment, the above-ground biomass of all grafts was partitioned into stem (including branches) and needles. Soil was carefully washed off roots to avoid breaking off fine roots. All plant material was oven-dried at 70 ºC for at least 72 h followed by dry weight determination. Root:shoot ratios were calculated for each graft. Leaf area was measured as total projected needle area for each of the ten grafts (pre-treatment samples) using a LI-3100C area meter (LI-COR Biosciences, Lincoln, NE, USA). We modelled the linear relationship between leaf area and dry mass for each genotype (see ‘Data analysis’ below).

### Gas exchange and chlorophyll fluorescence measurements

All gas exchange and chlorophyll fluorescence measurements were taken on fully hardened and expanded current-year needles from a lateral shoot adjacent to the defoliated portion of the crown, i.e. the branches from the upper crown that were closest to the defoliated lower half crown. Instant light–response curves were performed in a sub-sample of ten grafts to assess saturating levels of the photosynthetic photon flux density (*PPFD_sat_*) at the maximum apparent electron transport rate (*ETR*), using an Imaging-PAM chlorophyll fluorometer (M-series, Walz, Effeltrich, Germany) (Rascher *et al.*, 2000). The determined *PPFD_sat_* of 600 µmol m^–2^ s^–1^ was used in all subsequent gas exchange and chlorophyll fluorescence measurements. Hourly physiological measurements were performed from 08:00 h to 14.30 h (using 30 min at each time slot) in a sub-sample of ten grafts using a coupled chlorophyll fluorescence and gas exchange system (Imaging-PAM M-Series and GFS-3000, Walz, Effeltrich, Germany) at 400 ppm CO_2_ concentration, 20 ºC cuvette temperature, 60 % relative humidity and *PPFD_sat_*. We assessed differences between time slots for the parameters *ETR*, net photosynthetic rate (*A*) and stomatal conductance (*g*) by fitting a linear mixed model for each response variable, with ‘graft identity’ as random factor and ‘time slot’ as fixed factor (function lme, package nlme, Pinheiro *et al.*, 2018). Post-noon recordings (from 13.00 to 14.30 h) differed significantly from earlier recordings; therefore, subsequent measurements were performed between 08.00 h and 12.30 h.

To assess compensatory photosynthesis in the remaining upper half of the crown, we monitored the apparent *ETR* at *PPFDsat* using the Imaging-PAM fluorometer. To capture the short- and long-term effects of the defoliation on the remaining needles, we measured on four dates: pre-treatment assessment (8–9 August 2016, winter), 3 weeks (7–8 September 2016, spring), 3 months (15–16 November 2016, spring) and 1 year (14–15 August 2017, winter) after the first-year defoliation, and 2 months after the second-year defoliation (24 November 2017, spring). *A/C*_i_ curves were recorded in a sub-sample of grafts (*n* = 20 to assess first-year treatments, and *n* = 40 for second-year treatments) using the coupled fluorometer–gas exchange system to assess leaf area of needles inside the cuvette by means of chlorophyll fluorescence. *A/C*_i_ curves were measured on four different dates: before any treatment, 5 weeks and 1 year after first-year defoliation, and 2 months after the second-year defoliation (Fig. 1). Gas exchange rates were first recorded at a leaf chamber CO_2_ concentration (*C*_a_) of 400 ppm CO_2_, before *C*_a_ was stepwise reduced to 200, 100 and 40 ppm; subsequently, *C*_a_ was returned to 400 ppm and then increased to 600, 800, 1000, 1500 and 2000 ppm. All *A/C*_i_*i* curves were performed at 20 ºC cuvette temperature, 60 % relative humidity and *PPFDsat*. Individual response curves were completed within 30–40 min. *A/C*_i_ curves were fitted following an asymptotic function [Eqn (1)], analysing the following parameters: photosynthetic capacity (*A*_max_; upper asymptote), dark respiration (*A*_min_; lower asymptote) and the CO_2_ compensation point (*γ*), which is the CO_2_ concentration when the net photosynthetic rate equals 0. Values for *g* obtained in the first measurement of the *A/C*_i_ curves (i.e. at 400 ppm) were used to assess possible compensatory photosynthesis due to stomatal control.

$$\begin{array}{l} A=A_{max}\left(1-{\left(1-\frac{A_{min}}{A_{max}}\right)}^{1-\frac{C_{i}}{\gamma }}\right) \end{array}$$ (1)

### Non-structural carbohydrates

Sampling of needles for NSC analyses was performed on five different dates on all grafts to capture short- and long-term effects (Fig. 1): pre-treatment assessments (9 August 2016, winter), 1 month after first-year defoliation (12 September 2016, spring), 12 months after first-year defoliation (23 August 2017, winter), 13 months after the first-year defoliation (18 September 2017, spring) and 3 months after the second-year defoliation (5 December 2017, summer). Two fascicles from the upper half crown were sampled, chopped into approx. 3 mm long pieces into 5 mL tubes and immediately frozen in liquid nitrogen. Once frozen, samples were stored in a portable cooler during the sampling and later transferred to a –80 ºC freezer. Stem and root NSC sampling was performed on the same grafts and dates as biomass assessments, because the sampling implied the destruction of the stem and roots. Fine and coarse roots were sampled (approx. 50 % each), washed and rinsed. Two 2 mm thick stem sections were cut at 4 cm above the graft union. Hardwood and bark were discarded. Stem and root samples were each chopped into 2 mL tubes and frozen and stored as previously described. All NSC samples were freeze-dried overnight using a FreeZone Freeze Dry System (Model 7934037, Labconco, Kansas City, MO, USA). Needle samples were added to 4 mL of grinding metal balls (SPEX) and then ground to a fine powder using a GenoGrinder 2010 (SPEX SamplePrep, Stanmore, UK). Stem and root samples were ground in an impact-resistant 2 mL tube containing 2 mm glass beads and 2 mm yellow zirconium oxide beads (Lysing Matrix H, MP Biomedicals, Auckland, New Zealand) using a bead mill homogenizer (Omni bead ruptor 24, Omni International, Kennesay, GA, USA).

We used the high-throughput method presented by Ramirez *et al.* (2015). We applied their models to predict the NSC concentrations in our samples from the spectra measured in an FT-NIR analyser (Bruker MPA Multi Purpose FT-NIR Analyzer). Reflectance spectra were taken from 1300 to 2650 nm, at a resolution of 16 cm^–1^ and averaged over 32 scans. The estimation of sugar and starch contents followed the same reference (referred to as ‘all tissues’ models in Ramirez *et al.*, 2015). The range of both sugar and starch concentrations we obtained for our samples contained negative and close to zero values consistent with previous reports on *P. radiata* (Cranswick *et al.*, 1987). Due to the low NSC content of our samples, close to or below the detection limit of chemical methods, we shifted the values to a positive range to maintain the relative differences. The absolute value of the minimum NSC content was added to all observations.

### Data analysis

All statistical computations and graphics were performed using software R version 3.5.1 (R Core Team, 2017). A linear model was fitted to leaf area as the response variable, including leaf dry mass and genotype as predictors. The best-fit model (adjusted *R*^2^ = 0.88) was used to calculate leaf area from leaf mass data for the second and final harvests to assess canopy recovery from defoliation treatments.

To perform the gas exchange measurements, we first fitted light–response curves to determine the saturated light level of the grafts, which was used in all the measurements throughout the experiment. To assess the compensatory photosynthesis (hypothesis 1), we used the *A/C*_i_ curve-derived parameters, the net photosynthetic rate and the stomatal conductance. Both light–response curves and *A/C*_i_ curves were fitted by generalized non-linear least squares (gnls function, package nlme, Pinheiro *et al.*, 2018). For light–response curves, we used the Eilers–Peeters equation (Eilers and Peeters, 1988). Heteroscedasticity, suggested by the model diagnostic plots, was modelled using light intensity as a variance covariate. We used inverse interpolation to derive *PPFDsat* from the fitted values (light level at 90 % of *ETRmax*). For *A/C*_i_ curves, heteroscedasticity was modelled using the intercellular CO_2_ concentration as a variance covariate. We determined the upper and lower asymptote (*A*_max_ and *A*_min_), respectively. We used inverse interpolation to determine the CO_2_ compensation point. Effects of defoliation treatment and genotype on *g*, *A*_max_, *A*_min_ and the CO_2_ compensation point were analysed separately for each measurement point: 5 weeks and 1 year after the first defoliation, and 2 months after the second defoliation. Linear models were fitted, and optimal predictors were determined by backwards model selection comparing nested models using the Akaike information criterion (AIC). Effects of defoliation, genotype and their interaction on the *ETR* were assessed in the same way, but at four measuring dates: 3 weeks, 3 months and 1 year after the first defoliation, and 2 months after the second defoliation. In the case of *g*, as a response variable, we used linear mixed-effect models (lme function, package nlme, Pinheiro *et al.*, 2018) to include ‘time slot’ as a random effect, because *g* was the most sensitive variable to the measuring time.

To assess the impact of defoliation on growth (hypothesis 2), the effects of defoliation and genotype on *h*, *d*, above-ground biomass and root:shoot ratio were likewise analysed by fitting linear models, separately at two measuring dates: 1 year after the first-year defoliation and 3 months after the second-year defoliation. To analyse the impact of defoliation on NSC (hypothesis 3), stem and root soluble sugar and starch concentrations were analysed using linear mixed-effect models (lme function, package nlme, Pinheiro *et al.*, 2018) separately at the two sampling dates: 1 year after the first defoliation and 3 months after the second defoliation. In these models, ‘tissue’ (stem or root) was included as a random factor, and ‘genotype’ and ‘treatment’ and their interaction were considered as fixed factors. Needle sugar and starch concentrations were analysed on the same sampling dates. The effect of the first-year defoliation was assessed by a linear mixed model with ‘graft identity’ as a random factor instead, and ‘defoliation’, ‘genotype’ and their interaction, as well as ‘sampling date’ (1 month, 12 months and 13 months after first-year defoliation) as fixed factors. To analyse the effect of the 2 year defoliation treatments on the needle sugar and starch concentrations at the end of the experiment (3 months after the second defoliation), a linear model was fitted, with the explanatory variables ‘defoliation’, ‘genotype’ and their interaction. Model selection was based on the AIC. Post-hoc tests using Tukey contrasts were carried out using the emmeans function (package emmeans, Lenth, 2018).

## RESULTS

### Compensatory photosynthesis

**Fig. 2.**
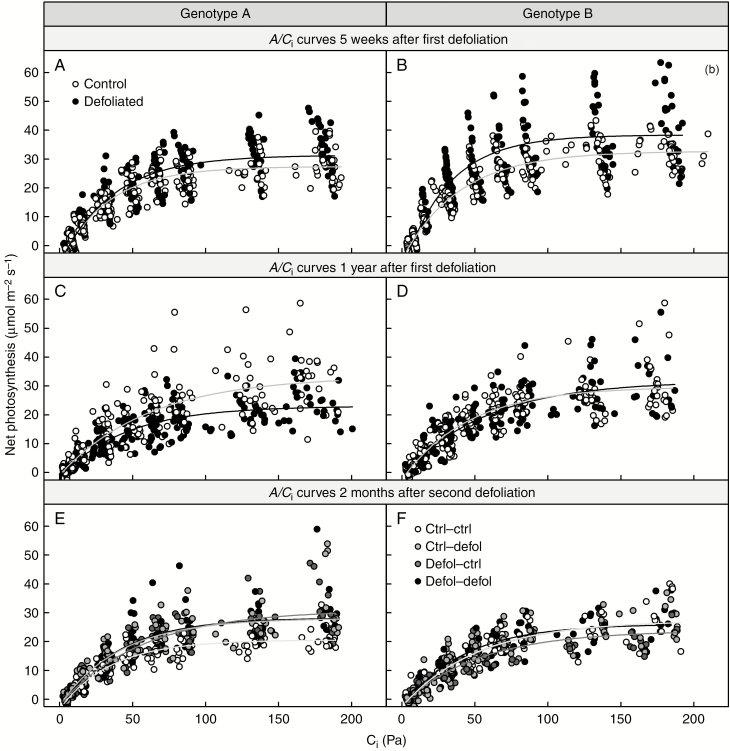
Response to defoliation of net photosynthesis as a function of intercellular CO_2_ concentration 5 weeks (A and B) and 1 year following first-year defoliation (C and D), and 2 months following second-year defoliation (E and F), for genotype A (A, C and E) and genotype B (B, D and F).

**Fig. 3.**
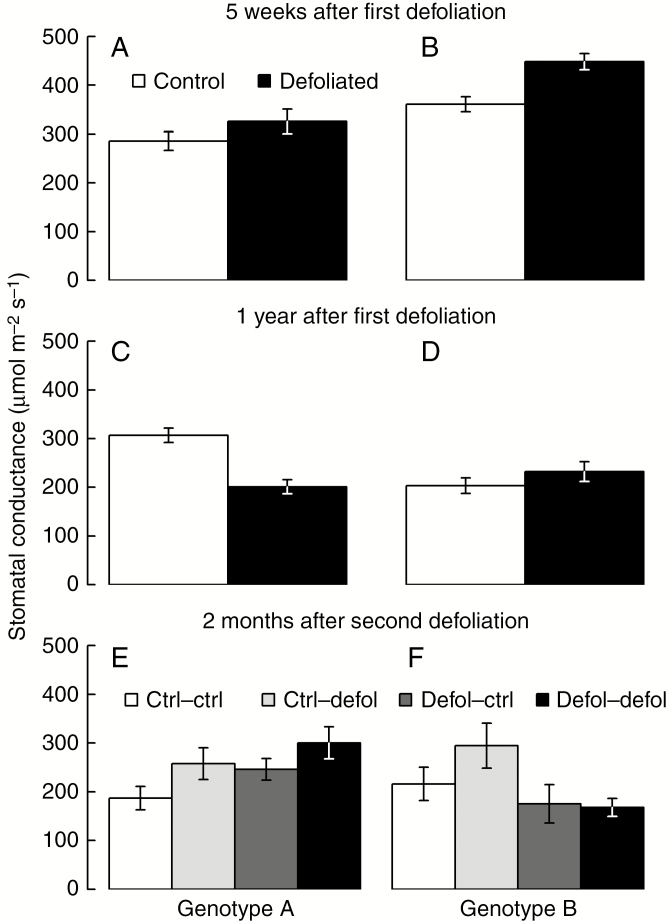
Response to defoliation of stomatal conductance 5 weeks (A and B) and 1 year following first-year defoliation (C and D), and 2 months following second-year defoliation (E and f).

No differences in the photosynthetic parameters measured in the remaining needles (*ETR* or *A/C*_i_ curves) were found between genotypes and defoliation treatments 3 months after the second defoliation (Figs 2 and 3; Supplementary data Table S1). Parameters derived from *A/C*_i_ curves (*A*_max_, *A*_min_ and CO_2_ compensation point) remained unaffected both by the first-year defoliation, 5 weeks and 1 year after the treatment, and by the second-year defoliation, 3 months after the treatment (Fig. 2; Supplementary data Table S1). Genotype B showed higher photosynthetic capacity than genotype A, 5 weeks after the first defoliation, regardless of the treatment (Fig. 2A, B; Supplementary data Table S1). Similarly, at the same time point, stomatal conductance was higher in genotype B than in genotype A (Fig. 3A, B; Supplementary data Table S1). One year after the first defoliation, no physiological impacts were detected apart from a reduction in stomatal conductance in genotype A compared with the genotype B and control grafts (Fig. 3C–F; Supplementary data Table S1).

### Growth and biomass

**Fig. 4.**
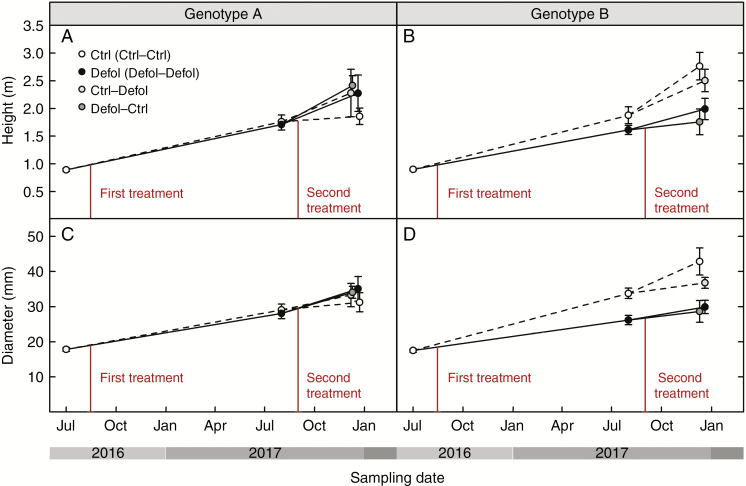
Response to defoliation of height (A and B) and diameter (C and D) for genotype A (A and C) and genotype B (B and D).

**Fig. 5.**
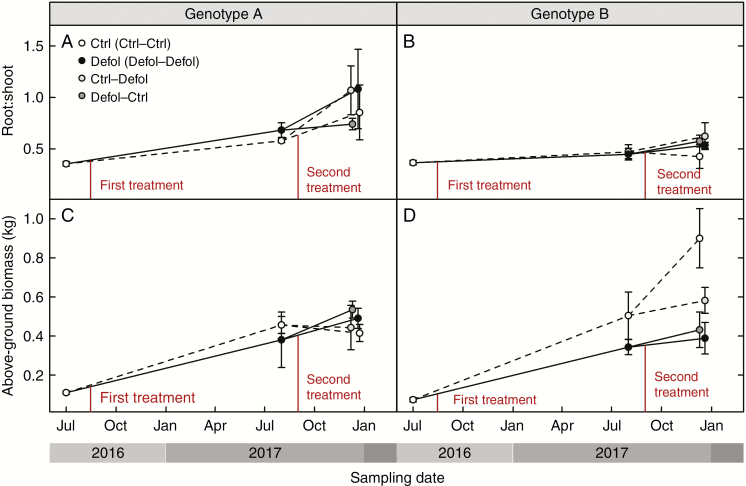
Response to defoliation of root:shoot ratio (A and B) and above-ground biomass including needle mass (C and D) for genotype A (A and C) and genotype B (B and D).

**Fig. 6.**
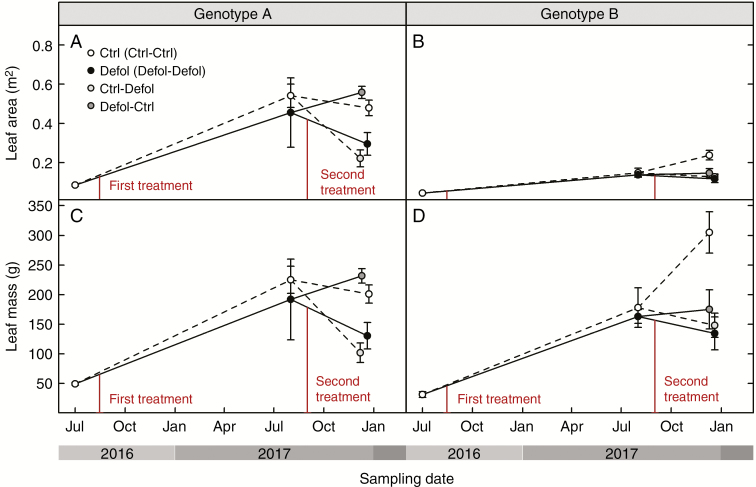
Response to defoliation of leaf area (A and B) and leaf mass (C and D) for genotype A (A and C) and genotype B (B and D). Leaf mass was directly measured. Leaf area was directly measured only before treatments, and then was predicted by leaf mass using regression equations.

Genotype A’s growth and biomass were not affected by any defoliation treatment at any time (Figs 4A, C and 5C; Supplementary data Table S2), except for leaf area and leaf mass which were reduced 3 months after a single defoliation treatment (second-year defoliation, Fig. 6A; Supplementary data Table S2c). This short-term effect was not detected 1 year after the first defoliation. In genotype B, 1 year after the first defoliation, a 23 % reduction in stem diameter was the only noticeable effect resulting from the experimental needle removal (Fig. 4D; Supplementary data Table S2). The second-year defoliation led to a further decrease in stem diameter in the previously defoliated genotype B grafts (32 % lower than control grafts, Fig. 4D; Supplementary data Table S2). In addition, height and total above-ground biomass were reduced in first-year defoliated grafts after the second growing season by 36 and 55 % in genotype B (Figs 4B and 5D). The root:shoot ratio remained unaffected by the treatments, but was significantly higher in genotype A compared with genotype B.

The correlation between leaf mass and leaf area was significant and varied among genotypes (significant leaf mass *×* genotype interaction, *L* = 5.66, d.f. = 1, *P* = 0.02). The best-fit model (adjusted *R*^2^ = 0.88) was used to estimate leaf area from leaf mass. Genotype A presented higher overall leaf area values than genotype B (Fig. 6A, B). Even though leaf mass did not differ between genotypes 1 year after defoliation, genotype B presented a lower leaf area, irrespective of the treatment (Fig. 6; Supplementary data Table S2). After the second growing season, genotype B grafts, which had undergone defoliation in the first year, presented lower leaf mass and leaf area compared with control trees. In contrast, genotype A defoliated grafts showed similar leaf mass and leaf area values to control grafts, 1 year after defoliation (Fig. 6; Supplementary data Table S2). Remarkably, while leaf area of genotype A control grafts was higher compared with that of genotype B, leaf mass was lower (Supplementary data Table S2).

### Non-structural carbohydrates

**Fig. 7.**
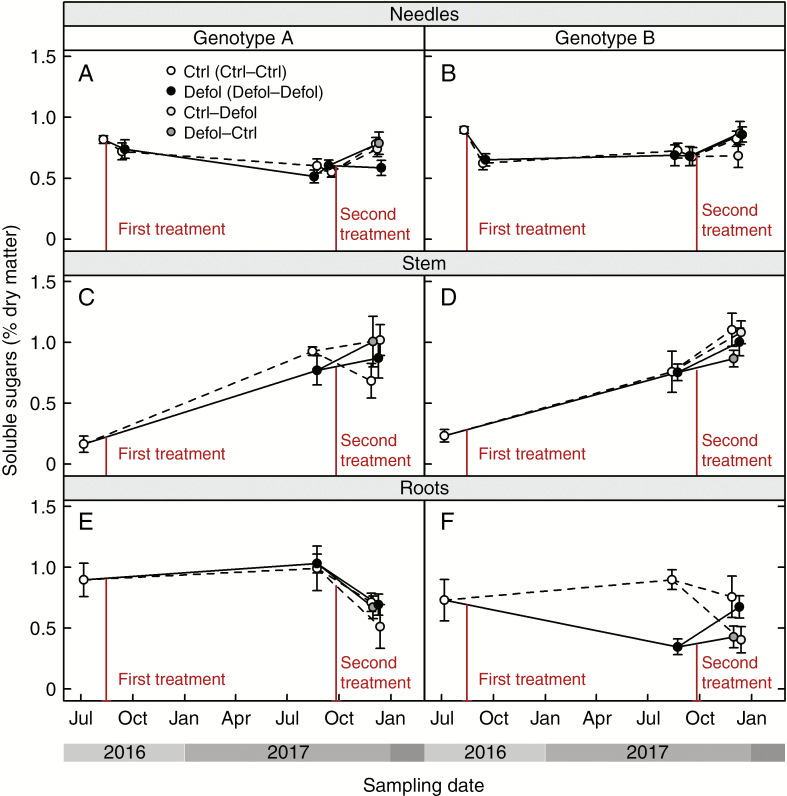
Response to defoliation of soluble sugar concentrations in needles (A and B), stem (C and D) and roots (E and F) for genotype A (A, C and E) and genotype B (B, D and F).

In genotype B, the first-year defoliation led to a decrease in sugar storage in roots after the first growing season, followed by a recovery of root sugar reserves over the second spring season (toward the end of the experiment; Fig. 7F; Supplementary data Table S3). However, this effect was not observed in genotype A grafts. Among the grafts that experienced two defoliation treatments, those of genotype A showed lower sugar concentrations in needles than those of genotype B (Fig. 7A, B; Supplementary data Table S3). Similar levels of sugar concentration in stems were found across genotypes and treatments. Irrespective of tissue type, starch content was similar among genotypes and remained unaffected by defoliation (Supplementary data Table S3).

## DISCUSSION

### Carbon assimilation under lower-crown defoliation

Our hypothesis of no or little compensatory photosynthesis in the remaining foliage following defoliation was confirmed, with no evidence of photosynthetic upregulation observed either biochemically or through stomatal control in response to lower-crown defoliation. This outcome contradicts earlier studies showing compensatory photosynthesis in both angiosperms (Eyles *et al.*, 2009; Quentin *et al*., 2010, 2012; Pinkard *et al.*, 2011; Barry and Pinkard 2013; Borzak *et al.*, 2017) and gymnosperms (Reich *et al.*, 1993; Vanderklein and Reich 1999; Little *et al.*, 2003), including *P. radiata* (Eyles *et al.*, 2011*a*). However, those studies focused on either upper- or whole-crown defoliation, mainly simulating insect herbivory. Unlike lower-crown defoliation, removal of upper-crown foliage increases light penetration to lower-crown leaves, enhancing the photosynthetic activity of residual leaves (Reich *et al.*, 1993). If the defoliation is severe enough, the source:sink ratio decreases as a recognized mechanism leading to photosynthetic upregulation (Wareing and Patrick, 1975; Lavigne *et al.*, 2001; Battaglia *et al.*, 2011). In our study, the first defoliation was apparently insufficient to change the source:sink ratio and cause any stimulatory effects on gas exchange in the remaining upper-crown needles. Also, the light conditions for upper-crown foliage remained unchanged with the removal of lower-crown leaves.

While neither genotype showed compensatory photosynthesis at any point, variation in the growth response to defoliation was observed between genotypes. Lower-crown defoliation had no effect on primary and secondary growth and NSC content in genotype A, indicating that it was not affected by C shortage. Plant canopies can present high leaf area index values beyond saturation with no further contribution to C assimilation (Thomas and Sadras, 2001; Weiner and Freckleton, 2010; Körner, 2018). Genotype A appeared to be saturated in terms of leaf area, even after defoliation. This finding suggests a redundancy of lower-crown foliage with regard to C assimilation in mature trees in dense evergreen forests or plantations, as their lower-crown foliage is mostly exposed to deep shade. In contrast, genotype B showed reduced growth and biomass accumulation 1 year following the first defoliation treatment (see next section). Intriguingly, few studies have also reported no effect of defoliation on photosynthesis, but have reported altered C allocation (Cerasoli *et al.*, 2004) and prioritization of C storage at the expense of growth (Wiley *et al.*, 2013). In genotype B, defoliation caused C shortage but no evident decrease in the source:sink ratio as no upregulation was detected. This could be explained by a reduction of growth-related sink demands, due to the winter period, which coincided with the defoliation-induced decrease in C uptake. Alternatively, the source:sink ratio might have decreased due to foliage removal when upper-crown leaves were photosynthesizing at maximum capacity and thus unable to respond to the increase in sink strength.

### Carbon allocation under lower-crown defoliation

Carbon shortage and changes in C allocation due to defoliation differed among genotypes. In genotype B, defoliation reduced both above-ground growth and sugar storage in roots. No such effects were observed in genotype A. Primary growth reduction (reduced height increase) occurred only after the second growing season in response to the first defoliation, regardless of the second-year treatment (Fig. 4B; Supplementary data Table S2). Impacts on secondary growth (radial growth) became apparent earlier than on primary growth: 1 year after the first defoliation in genotype B (Fig. 4D; Supplementary data Table S2). The second defoliation in first-year control plants did not have an impact on diameter 3 months after the treatment (Fig. 4D; Supplementary data Table S2). Potential impacts on secondary growth may only emerge at a later date, as suggested by the effects found 1 year after the first defoliation. Defoliation-induced reductions in radial growth have been reported to range from 0 to 37 % in 50 % defoliation treatments, and from 40 to 88 % reduction in complete defoliation treatments (Little *et al.*, 2003; Wills *et al.*, 2004; Saffell *et al.*, 2013; Jacquet *et al.*, 2014; Piper *et al.*, 2015; Puri *et al.*, 2015; Wiley *et al.*, 2017*a*). Our results further support the idea that radial growth is more sensitive to defoliation than vertical extension (Rook and Whyte, 1976; Quentin *et al.*, 2010; Jetton and Robison, 2014; Lombardero *et al.*, 2016; Borzak *et al.*, 2017). The extent to which growth in diameter and height is reduced may vary depending on: the intensity of defoliation (height was only altered by severe defoliation, Rook and Whyte, 1976); upregulation of photosynthesis in remaining leaves (Eyles *et al.*, 2011*b*; Quentin *et al.*, 2012); competing sink strength of C pools (Palacio *et al.*, 2012); time of the defoliation with respect to leaf phenology (Chen *et al.*, 2017); and portion of affected crown (see below).

The defoliation treatments in our experiment imposed no apparent C shortage in genotype A, and caused a growth reduction in genotype B. Previous defoliation studies demonstrated that the impact on growth is negligible below a given threshold which can be variable across experiments (Rapley *et al.*, 2009; Jetton and Robison 2014), including conifer species (Vanderklein and Reich, 1999; Jacquet *et al.*, 2014; Deslauriers *et al.*, 2015; Lombardero *et al.*, 2016). Our findings suggest that this threshold may vary among genotypes, possibly driven by differences in leaf traits. Interestingly, genotype A presented higher leaf area at similar values of leaf mass, i.e. higher specific leaf area (*SLA*), which has been associated with a more efficient use of resources (Hodgkinson, 1974; Alderfer and Eagles, 1976; Niinemets, 1999; Warren and Adams, 2000), which might have contributed to the divergent defoliation impacts. Even though a similar proportion of needles were artificially removed in both genotypes, lower *SLA* saw greater leaf mass removed from genotype B, which was later most affected by C shortage. Leaves with lower *SLA* values have higher C content (Bresinsky *et al.*, 2013), and remain photosynthetically active for a longer period of time to compensate for their high C cost (Reich *et al.*, 1992; Bresinsky *et al.*, 2013). Hence, defoliation in genotype B, which showed lower *SLA* values at the beginning of the experiment, meant a more dramatic decrease in photosynthetically active tissue, which is consistent with the observed post-defoliation C shortage. This hypothesis is supported by higher photosynthetic capacity 5 weeks after first-year treatments in genotype B, regardless of the defoliation treatment (Supplementary data Table S1).

In our experiment, defoliation resulted in a preferential shift in new biomass production towards foliage regeneration to restore leaf area and root:shoot ratios (Figs 5A, B and 6A, B), as root biomass was not significantly different between treatments (results not shown). Similar biomass allocation patterns have been previously observed to maintain leaf area (Barry and Pinkard 2013) and root:shoot ratios after defoliation (Wareing and Patrick, 1975; Krause and Raffa, 1992; Reich *et al.*, 1993; Schmid *et al.*, 2017). However, at the end of our experiment, genotype B grafts that were defoliated at least once presented lower leaf mass than control grafts (Fig. 6D; Supplementary data Table S2). This observed long-term decrease in C allocation to leaf area regeneration, together with the sustained decrease in above-ground growth (Figs 4B, D and 5D; Supplementary data Table S2), could have occurred at the expense of reduced translocation of soluble sugars to roots in first-year defoliated grafts (Fig. 7F; Supplementary data Table S3). Thus, in the short term, the first-year defoliation in genotype B would have led to crown formation and restoration of the root:shoot ratio at the expense of sugar content in roots, while in the long term, translocation of soluble sugars to roots was prioritized over above-ground growth. This is consistent with transient NSC decreases following defoliation previously reported (Palacio *et al*., 2008, 2012; Saffell *et al.*, 2013) and reduced C reserves in the roots (Vanderklein and Reich, 1999; Quentin *et al*., 2011; Landhausser and Lieffers, 2012; Chen *et al.*, 2017), suggesting that restoring NSC reserves takes precedence over growth.

The transient decrease in NSC storage seemed to have had no or little impact on tree C relations, as starch concentrations across tissues and genotypes remained unaffected by any defoliation treatment. Defoliated grafts may have either actively maintained similar levels of starch to control grafts, or met sink demands by the C assimilated by surviving foliage, leaving C storage unaltered. Starch pools were probably not mobilized in the recovery process either, suggesting that either NSC remobilization and transport may be impaired or slower than needed, or that starch may contribute to maintain a baseline level of NSC (Wiley and Helliker, 2012; but see Wiley *et al.*, 2017*b*). However, we cannot rule out the possibility of remobilization and rapid replenishment of starch reserves through the C-fixing ability of the remaining foliage. Indeed, starch concentrations appeared to be already relatively low in our grafts from the start. This is consistent with previous reports for New Zealand-grown *P. radiata*, as a result of its year-round, continuous sink activity in New Zealand (Cranswick *et al.*, 1987). A 2 year artificial defoliation experiment in *Pinus resinosa* and *Larix leptolepis*, similar to the one presented here, also showed a preferential, but small, reduction in sugar reserves rather than starch upon defoliation (Vanderklein and Reich, 1999). Starch concentrations have been shown to be well correlated with the ability of trees to survive defoliation events (Webb, 1981) which explains why plants tend to maintain starch reserves unless facing severe stress.

Our defoliation treatments were applied during late winter to mimic the impacts of RNC disease. This was before the critical period of leaf formation in spring, and had little impact on NSC reserves. It has been shown that defoliation occurring before the regeneration of foliage causes less growth retardation and has a lower impact on C reserves than defoliation late in the growing season (Rook and Whyte, 1976; Barnes and Edwards, 1980; Chen *et al.*, 2017). Thus, the pronounced seasonality of the RNC disease could have a lower impact on tree growth than pathogens affecting canopy development during spring and summer.

### The enigmatic role of the lower crown

Our findings put forward the specific proportion of the crown affected by defoliation as a new factor to consider within the framework of tree C dynamics. Defoliation treatments performed in our experiment aimed at mimicking RNC disease, removing 1-year-old and older needles from the bottom half of the crown. In genotype A, the removal of this portion of the crown did not impose any C shortage on the plant. The contribution of older, lower-crown needles to whole-tree C assimilation is smaller than that of current-year needles in the upper crown (Leuning *et al.*, 1991; Bresinsky *et al.*, 2013). Therefore, artificial removal of those leaves would have little impact on tree C balance. Why would a tree spend C and energy to maintain this ‘extra’ foliage? The importance of the lower-crown foliage is certainly controversial. Moreira *et al.* (2012) found that *P. radiata* needles had better developed chemical defences, both resin and phenolic compounds, in the basal parts of the plant, despite their apparent lower value measured by lower N concentrations. This outcome is contrary to the optimal defence theory which states that plants have higher levels of chemical defences in the tissues that are contributing more to plant fitness (Zangerl and Bazzaz 1992). This well-defended lower-crown foliage could be a buffer to pathogen infection of the upper crown, which contributes most to whole-plant C assimilation. It has been suggested that excess foliage can also serve as nutrient storage that can be translocated to younger foliage (Hirose, 2012; Körner, 2018). Many evergreen conifers store N and NSC preferentially in the old foliage (Millard *et al.*, 2001; Li *et al.*, 2002).

The importance of old needles was also highlighted by Little *et al.* (2003) who showed that, across the whole crown, the removal of 1-year-old and older foliage had a greater impact on growth than the removal of current-year foliage in balsam fir. Those findings were consistent with observations in the field suggesting slower tree recovery after defoliation by balsam fir sawfly (removal of 1-year-old and older foliage) than by spruce budworm (removal of current-year foliage; Piene *et al.*, 2001). Another artificial defoliation experiment comparing the effect of different needle cohorts across the whole crown in *P. radiata* found that the removal of only current-year needles had significantly less impact on diameter and height growth than the removal of 1- to 3-year-old needles (Rook and Whyte, 1976). Further, even the removal of current- and 1-year-old needles had less impact on growth than defoliation of 1- to 3-year-old needles (Rook and Whyte, 1976). This is consistent with the remarkable impact of removal of stored N on growth in the genus *Pinus* (Millard *et al.*, 2001), with 31–57 % of N for new shoot growth remobilized from storage in *P. radiata*, whose previous-year needles are the main site of N storage (Nambiar and Fife, 1987; Millard and Grelet, 2010).

There are very few studies on the impact of foliar pathogen-induced defoliation on C assimilation and allocation. The Swiss needle cast disease and its impact on Douglas fir trees, caused by the ascomycete fungus *Nothophaeocryptopus gaeumannii*, has been thoroughly studied (Manter *et al.*, 2003; Saffell *et al*., 2013, 2014). In contrast to the majority of foliar pathogens, *N. gaeumannii* presents higher rates of infection in the upper than in the lower crown (Shaw *et al.*, 2014). Typical foliar diseases in pine forest systems, such as RNC and *Dothistroma* needle blight, are dispersed by rain splash and fog, and require high needle wetness (Bulman *et al.*, 2013; Dick *et al.*, 2014). Thus, those pathogens typically start infection from the bottom canopy, which may spread up to the top under conducive environmental conditions. Although growth losses have been documented in a few studies (Krause and Raffa, 1992; Haugen and Ostry, 2013; Saffell *et al.*, 2014; Mullett and Brown, 2018), very little is known about the mechanisms leading to reduced growth rates. A recent study on the infection of *Pinus nigra* by *Dothistroma septosporum* showed a correlation between infection and reduction in shoot length, which may compromise the development of photosynthetic capacity of the tree and contribute to reduced volume growth (Mullett and Brown, 2018). Krause and Raffa (1992) also reported reduced shoot production following defoliation by the foliar pathogen *Mycosphaerella laricinia* on *Larix* sp., suggesting a long-term impact on growth and survival of larch through reduction of its optimum photosynthetic area and biomass productivity. In the same study, the comparison between pathogen- and insect-induced defoliation elucidated that complete pathogen-induced defoliation causes more serious damage to the tree. Indeed, while insect attacks cause fast defoliation (only minutes), pathogen-infected leaves may remain on shoots for longer (several weeks) (Krause and Raffa, 1992), becoming carbon sinks until they are shed (Manter *et al.*, 2003). Although we have not accounted for this effect in this study, the defence process associated with pathogenic infection and defoliation appears to be longer and more C costly compared with insect attack. Moreover, if the disease progresses to the upper crown causing complete defoliation, its impact on tree productivity becomes larger (Rook and Whyte, 1976; Krause and Raffa, 1992).

### Conclusions

The physiological response of *P. radiata* to lower-crown defoliation is genotype specific. Even though the two studied genotypes were similarly susceptible to the RNC disease, they showed different tolerance to defoliation. This finding highlights the importance of factors other than disease susceptibility, such as leaf functional traits, for the resilience and productivity of *P. radiata* stands and has far-reaching implications for future forest disease management.

Our experiment was a first approach to understand the role and function of the lower crown, as target foliage by pathogens, and raises the question of the ecological role of mild foliar pathogenic infections. Pathogen-induced defoliations have not been given sufficient attention because they are perceived as a non-lethal biotic stress. However, defoliations have been broadly recognized to be a predisposing event for trees to enter a spiral of decline when other biotic or abiotic stress episodes concur (Manion, 2003; Oliva *et al.*, 2016). Further research is needed to approach the remaining open questions. Are pathogenic outbreaks more damaging than insect attacks when the whole crown is affected, justifying higher investment in chemical compounds in the lower crown for an early stop of infection progress? What are the thresholds for pathogen-induced defoliation in terms of tree productivity and survival? Does the lower crown have a function of buffering pathogenic infection to protect highly productive foliage, but providing a substrate for pathogens to maintain the pathogen population so that it controls competing vegetation? A common framework bringing together different disciplines – plant pathology, tree physiology and chemistry – is needed to fully address the fundamental ecological questions raised here.

## SUPPLEMENTARY DATA

Supplementary data are available online at https://academic.oup.com/aob and consist of the following. Table S1: photosynthetic parameters for control and defoliated grafts of genotypes A and B. Table S2: height, diameter, root:shoot ratio, woody above-ground biomass, leaf area and leaf biomass for control and defoliated grafts of genotypes A and B. Table S3: soluble sugar and starch concentrations for control and defoliated grafts of genotypes A and B.

mcaa013_suppl_Supplementary_Table_S1

mcaa013_suppl_Supplementary_Table_S2

mcaa013_suppl_Supplementary_Table_S3

## FUNDING

The authors are indebted to The Forest Owners Association, New Zealand, and Ministry of Business Innovation and Employment (C04X1305), New Zealand, who provided the funding for this work.
