# Supplementary material for: No carbon limitation after lower crown loss in Pinus radiata
Source: Ann Bot. 2020 Jan 28;125(6):955–67. doi: 10.1093/aob/mcaa013 (PMC7218809; doi:10.1093/aob/mcaa013)
Supplement: mcaa013_suppl_Supplementary_Table_S1 [file mcaa013_suppl_supplementary_table_s1.docx]

Supplementary material

Table S1. Photosynthetic parameters for control (Ctrl) and defoliated (Defol) grafts of genotypes A and B, five weeks and one year after the first-year defoliation treatment, and three months after the second-year defoliation treatment. Number in parenthesis indicate standard errors. Different letters indicate statistically significant differences between groups at P = 0.05 on a multiple comparison procedure using Tukey.

|  | **Genotype A** | | | | **Genotype B** | | | |
| --- | --- | --- | --- | --- | --- | --- | --- | --- |
| **Five weeks after** | **Ctrl** | | **Defol** | | **Ctrl** | | **Defol** | |
| *A*_max_ | 27.49 (2.50) ^a^ | | 30.81 (2.50) ^a^ | | 33.93 (2.58) ^b^ | | 37.26 (2.67) ^b^ | |
| *A*_min_ | -9.03 (1.54) ^a^ | | -6.81 (1.61) ^a^ | | -6.80 (1.54) ^a^ | | -11.25 (1.70) ^a^ | |
| CO_2_ comp point | 6.05 (0.78) ^a^ | | 6.25 (0.78) ^a^ | | 6.04 (0.81) ^a^ | | 6.24 (0.83) ^a^ | |
| *g* | 273.83 (26.83) ^a^ | | 373.83 (27.73) ^a^ | | 330.90 (27.73) ^b^ | | 430.88 (28.68) ^b^ | |
| **One year after** | **Ctrl** | | **Defol** | | **Ctrl** | | **Defol** | |
| *Amax* | 35.59 (4.09) ^a^ | | 27.96 (4.25) ^a^ | | 35.59 (4.09) ^a^ | | 28.40 (4.25) ^a^ | |
| *Amin* | -1.57 (0.68) ^a^ | | -1.65 (0.63) ^a^ | | -3.03 (0.65) ^a^ | | -3.10 (0.68) ^a^ | |
| CO_2_ comp point | 2.55 (0.70) ^a^ | | 2.78 (0.64) ^a^ | | 3.19 (0.67) ^a^ | | 3.42 (0.70) ^a^ | |
| *g* | 301.33 (35.69) ^a^ | | 191.04 (38.15) ^b^ | | 212.16 (35.69) ^a^ | | 234.53 (35.69) ^a^ | |
| **Three months after** | **Ctrl-Ctrl** | **Ctrl-Defol** | **Defol-Ctrl** | **Defol-Defol** | **Ctrl-Ctrl** | **Ctrl-Defol** | **Defol-Ctrl** | **Defol-Defol** |
| *A*_min_ | 24.68 (3.48) ^a^ | 29.73 (3.48) ^a^ | 29.91 (3.66) ^a^ | 29.16 (3.48) ^a^ | 22.12 (3.48) ^a^ | 27.17 (3.48) ^a^ | 27.35 (3.99) ^a^ | 26.60 (3.48) ^a^ |
| *A*_min_ | -3.47 (0.62) ^a^ | -2.63 (0.62) ^a^ | -2.46 (0.66) ^a^ | -3.19 (0.62) ^a^ | 3.73 (0.62) ^a^ | -2.90 (0.62) ^a^ | -2.73 (0.72) ^a^ | -3.46 (0.62) ^a^ |
| CO_2_ comp point | 4.66 (0.75) ^a^ | 3.69 (0.75) ^a^ | 3.43 (0.79) ^a^ | 3.98 (0.75) ^a^ | 5.54 (0.75) ^a^ | 4.57 (0.75) ^a^ | 4.31 (0.86) ^a^ | 4.86 (0.74) ^a^ |
| *g* | 207.73 (43.79) ^a^ | 291.42 (43.79) ^a^ | 237.35 (46.03) ^a^ | 242.32 (43.79) ^a^ | 176.61 (43.79) ^a^ | 260..31 (43.78) ^a^ | 206.24 (50.23) ^a^ | 211.21 (43.79) ^a^ |
