# Supplementary material for: No carbon limitation after lower crown loss in Pinus radiata
Source: Ann Bot. 2020 Jan 28;125(6):955–67. doi: 10.1093/aob/mcaa013 (PMC7218809; doi:10.1093/aob/mcaa013)
Supplement: mcaa013_suppl_Supplementary_Table_S2 [file mcaa013_suppl_supplementary_table_s2.docx]

Supplementary material

Table S2. Height (*h*, m), diameter (*d*, mm), root:shoot ratio, woody aboveground biomass (abvgr biomass, kg), leaf area (*LA*, m^2^), and leaf biomass (*LM*, g) for control (Ctrl) and defoliated (Defol) grafts of genotypes A and B, one year after the first-year defoliation treatment, and three months after the second-year defoliation treatment. Number in parenthesis indicate standard errors. Different letters indicate statistically significant differences between groups at P = 0.05 on a multiple comparison procedure using Tukey.

|  | Genotype A | | | | Genotype B | | | |
| --- | --- | --- | --- | --- | --- | --- | --- | --- |
| **One year after** | **Control** | | **Defoliated** | | **Control** | | **Defoliated** | |
| *h* | 1.82 (0.11) ^a^ | | 1.65 (0.11) ^a^ | | 1.83 (0.10) ^a^ | | 1.66 (0.10) ^a^ | |
| *d* | 29.0 (1.5) ^a^ | | 28.5 (1.5) ^a^ | | 33.7 (1.4) ^b^ | | 26.1 (1.4) ^a^ | |
| Root:shoot | 0.61 (0.05) ^a^ | | 0.63 (0.06) ^a^ | | 0.45 (0.05) ^b^ | | 0.47 (0.05) ^b^ | |
| Abvgr biomass | 2.07 (0.50) ^a^ | | 2.08 (0.55) ^a^ | | 2.57 (0.50) ^a^ | | 2.59 (0.52) ^a^ | |
| *LA* | 0.46 (0.06) ^a^ | | 0.55 (0.06) ^a^ | | 0.10 (0.06) ^b^ | | 0.19 (0.06) ^b^ | |
| *LM* | 189.5 (25.6) ^a^ | | 230.6 (27.9) ^a^ | | 153.2 (25.3) ^a^ | | 194.3 (26.4) ^a^ | |
| **Three months after** | **Ctrl-Ctrl** | **Ctrl-Defol** | **Defol-Ctrl** | **Defol-Defol** | **Ctrl-Ctrl** | **Ctrl-Defol** | **Defol-Ctrl** | **Defol-Defol** |
| *h* | 1.86 (0.26) ^a^ | 2.28 (0.26) ^a^ | 2.41 (0.26) ^a^ | 2.27 (0.26) ^a^ | 2.76 (0.26) ^b^ | 2.51 (0.24) ^ab^ | 1.76 (0.24) ^a^ | 1.99 (0.24) ^ab^ |
| *d* | 31.2 (2.9) ^a^ | 33.3 (2.9) ^a^ | 34.1 (2.9) ^a^ | 35.1 (2.9) ^a^ | 42.9 (2.9) ^b^ | 36.8 (2.6) ^ab^ | 28.7 (2.6) ^a^ | 29.9 (2.6) ^a^ |
| Root:shoot | 0.83 (0.13) ^a^ | 1.04 (0.13) ^a^ | 0.85 (0.13) ^a^ | 0.98 (0.15) ^a^ | 0.45 (0.13) ^b^ | 0.65 (0.13) ^b^ | 0.46 (0.13) ^b^ | 0.60 (0.14) ^b^ |
| Abvgr biomass | 1.99 (0.49) .41^a^ | 0.92 (0.49) ^a^ | 1.20 (0.49) ^a^ | 0.93 (0.56) ^a^ | 3.14 (0.49) ^b^ | 2.07 (0.49) ^b^ | 2.35 (0.50) ^b^ | 2.07 (0.51) ^b^ |
| *LA* | 0.48 (0.03) ^a^ | 0.22 (0.03) ^b^ | 0.56 (0.03) ^a^ | 0.30 (0.03) ^b^ | 0.24 (0.03) ^c^ | 0.13 (0.03) ^bc^ | 0.15 (0.03) ^c^ | 0.12 (0.03) ^c^ |
| *LM* | 201.0 (24.1) ^ab^ | 101.8 (24.1) ^c^ | 231.6 (24.1) ^b^ | 130.4 (24.1) ^ac^ | 305.1 (24.1) ^d^ | 148.1 (24.1) ^c^ | 175.2 (24.1) ^bc^ | 134.3 (24.1) ^ac^ |
