# Supplementary material for: No carbon limitation after lower crown loss in Pinus radiata
Source: Ann Bot. 2020 Jan 28;125(6):955–67. doi: 10.1093/aob/mcaa013 (PMC7218809; doi:10.1093/aob/mcaa013)
Supplement: mcaa013_suppl_Supplementary_Table_S3 [file mcaa013_suppl_supplementary_table_s3.docx]

Supplementary material

Table S3. Soluble sugar and starch concentrations (% dry matter) for control (Ctrl) and defoliated (Defol) grafts of genotypes A and B, one year after the first-year defoliation treatment, and three months after the second-year defoliation treatment, in roots and stem; and in needles during three sampling dates following first year defoliation and final sampling after second-year defoliation. Number in parenthesis indicate standard errors. Different letters indicate statistically significant differences between groups at P = 0.05 on a multiple comparison procedure using Tukey.

|  | **Genotype A** | | | | | | | | | | | | **Genotype B** | | | | | | | | | | | |
| --- | --- | --- | --- | --- | --- | --- | --- | --- | --- | --- | --- | --- | --- | --- | --- | --- | --- | --- | --- | --- | --- | --- | --- | --- |
| **Tissue** | **ROOT** | | | | | | **STEM** | | | | | | **ROOT** | | | | | | **STEM** | | | | | |
| **One year after** | **Ctrl** | | | **Defol** | | | **Ctrl** | | | **Defol** | | | **Ctrl** | | | **Defol** | | | **Ctrl** | | | **Defol** | | |
| Sugar | 0.99 (0.12) ^a^ | | | 1.03 (0.13) ^a^ | | | 0.93 (0.12) ^a^ | | | 0.77 (0.12) ^a^ | | | 0.90 (0.12) ^a^ | | | 0.35 (0.16) ^bc^ | | | 0.76 (0.12) ^a^ | | | 0.75 (0.12) ^ac^ | | |
| Starch | 1.35 (0.27) ^a^ | | | 1.55 (0.30) ^a^ | | | 1.29 (0.28) ^a^ | | | 1.49 (0.27) ^a^ | | | 1.49 (0.28) ^a^ | | | 1.69 (0.32) ^a^ | | | 1.43 (0.27) ^a^ | | | 1.63 (0.28) ^a^ | | |
|  | Ctrl | | | Defol | | | Ctrl | | | Defol | | | Ctrl | | | Defol | | | Ctrl | | | Defol | | |
| **Three months after** | Ctrl | Defol | | Ctrl | | Defol | Ctrl | Defol | | Ctrl | | Defol | Ctrl | Defol | | Ctrl | | Defol | Ctrl | Defol | | Ctrl | | Defol |
| Sugar | 0.66 (0.12)^a^ | 0.51 (0.12)^a^ | | 0.71 (0.11)^a^ | | 0.71 (0.11)^a^ | 0.72 (0.12)^ab^ | 1.00 (0.12)^b^ | | 1.00 (0.11)^bc^ | | 0.87 (0.11)^ab^ | 0.81 (0.12)^ac^ | 0.41 (0.11)^a^ | | 0.39 (0.11)^b^ | | 0.66 (0.11)^a^ | 1.07 (0.12)^c^ | 1.10 (0.10)^bc^ | | 0.87 (0.11)^c^ | | 1.00 (0.11)^bc^ |
| Starch | 1.39 (0.09)^a^ | 1.19 (0.09)^a^ | | 1.29 (0.09)^a^ | | 1.29 (0.09)^a^ | 3.68 (0.09)^b^ | 3.48 (0.09)^b^ | | 3.59 (0.09)^b^ | | 3.59 (0.09)^b^ | 1.41 (0.09)^a^ | 1.21 (0.09)^a^ | | 1.31 (0.09)^a^ | | 1.31 (0.09)^a^ | 3.70 (0.09)^b^ | 3.50 (0.09)^b^ | | 3.61 (0.09)^b^ | | 3.61 (0.09)^b^ |
|  | **Genotype A** | | | | | | | | | | | | **Genotype B** | | | | | | | | | | | |
| **NEEDLES** | 1 month after | | | | 12 months after | | | | 13 months after | | | | 1 month after | | | | 12 months after | | | | 13 months after | | | |
| 1st year treatment | Ctrl | | Defol | | Ctrl | | Defol | | Ctrl | | Defol | | Ctrl | | Defol | | Ctrl | | Defol | | Ctrl | | Defol | |
| Sugar | 0.67 (0.08) ^a^ | | 0.65 (0.08) ^a^ | | 0.70 (0.10) ^a^ | | 0.68 (0.10) ^a^ | | 0.61 (0.09) ^a^ | | 0.59 (0.08) ^a^ | | 0.65 (0.07) ^a^ | | 0.62 (0.07) ^a^ | | 0.68 (0.10) ^a^ | | 0.65 (0.10) ^a^ | | 0.59 (0.08) ^a^ | | 0.56 (0.08) ^a^ | |
| Starch | 2.05 (0.11) ^a^ | | 2.10 (0.11) ^a^ | | 2.18 (0.14) ^a^ | | 2.23 (0.14) ^a^ | | 2.08 (0.12) ^a^ | | 2.14 (0.12) ^a^ | | 1.88 (0.10) ^a^ | | 1.93 (0.10) ^a^ | | 2.01 (0.14) ^a^ | | 2.06 (0.15) ^a^ | | 1.91 (0.10) ^a^ | | 1.97 (0.10) ^a^ | |
| 1st year treatment | **Ctrl** | | | | | | **Defol** | | | | | | **Ctrl** | | | | | | **Defol** | | | | | |
| 2nd year treatment | **Ctrl** | | | **Defol** | | | **Ctrl** | | | **Defol** | | | **Ctrl** | | | **Defol** | | | **Ctrl** | | | **Defol** | | |
| Sugar | 0.78 (0.08) ^a^ | | | 0.74 (0.08) ^a^ | | | 0.79 (0.08) ^a^ | | | 0.58 (0.08) ^a^ | | | 0.82 (0.08) ^ab^ | | | 0.68 (0.07) ^ab^ | | | 0.87 (0.07) ^ab^ | | | 0.86 (0.07) ^b^ | | |
